# Fever of Unknown Origin in Myelodysplastic Syndrome: Clinical and Laboratory Features of a Chinese Cohort

**DOI:** 10.3390/jcm15218249

**Published:** 2026-10-23

**Authors:** Huiting Liu, Lifan Zhang, Miao Chen, Baotong Zhou, Hongwei Fan, Taisheng Li, Wei Cao

**Affiliations:** 1 Department of Infectious Diseases, Peking Union Medical College Hospital, Chinese Academy of Medical Science and Peking Union Medical College, No. 1 Shuaifuyuan, Wangfujing, Dongcheng District, Beijing 100730, China; 2 Department of Hematology, Peking Union Medical College Hospital, Chinese Academy of Medical Science and Peking Union Medical College, Beijing 100730, China

**Keywords:** fever of unknown origin, myelodysplastic syndrome, treatment response, anti-inflammatory therapy

## Abstract

**Background**: Fever of unknown origin (FUO) is clinically challenging in the setting of myelodysplastic syndrome (MDS). This study aimed to describe the clinical and laboratory features of patients with MDS and FUO and to explore factors associated with in-hospital fever resolution. **Methods**: We conducted a retrospective, single-center observational study of hospitalized patients with MDS and FUO at Peking Union Medical College Hospital, China, between January 2013 and December 2022. Patients were categorized into treatment-responsive and treatment-unresponsive groups based on fever outcomes after treatment. Demographic, clinical and laboratory features were collected and compared between the two groups. **Results**: A total of 98 patients were screened and 89 were included in the study. In approximately 80% of patients, FUO presented as the initial manifestation of MDS. Over half of the patients had high or very high risk per the revised International Prognostic Scoring System (IPSS-R), and complex karyotypes were observed in 25.8% of patients. MDS subtype distribution differed significantly between the groups (*p* = 0.027). Lower-blast MDS (MDS-LB) was more common in the treatment-responsive group (70.3% vs. 48.1%), whereas increased-blast MDS (MDS-IB) was more frequent in the treatment-unresponsive group (16.2% vs. 42.3%). Mucocutaneous lesions were more frequent among treatment-responsive patients (54.1% vs. 21.2%; *p* = 0.002). A higher percentage of CD8+CD38+ T-cells was associated with favorable treatment response (66.7 [60.4,80.6] vs. 47.4 [38.5,57.7]; *p* = 0.003). In addition, use of glucocorticoids and immunosuppressants was significantly associated with fever resolution (*p* = 0.004 and *p* = 0.043, respectively). **Conclusions**: In this selected cohort, MDS patients with FUO were predominantly at high risk and carried unfavorable prognostic factors. Mucocutaneous presentation and elevated CD8+CD38+ T-cell proportions were associated with in-hospital fever improvement. The association between anti-inflammatory treatment and fever resolution suggests a possible immune-mediated contribution to fever in these patients. As no non-MDS FUO comparison group was included and long-term outcomes were not available, these findings should be regarded as descriptive and hypothesis-generating.

## 1. Introduction

Myelodysplastic syndrome (MDS) is a heterogeneous group of clonal hematopoietic disorders characterized by ineffective hematopoiesis, peripheral cytopenia, and an increased risk of progression to acute myelogenous leukemia [1]. In clinical practice, fever is a common symptom in patients with MDS and has traditionally been attributed to infectious complications secondary to neutropenia or to leukemic transformation. However, accumulating clinical observations have revealed that a subset of MDS patients present with persistent fever even in the absence of identifiable infection, suggesting that the febrile episodes may be driven by immune dysregulation and systemic inflammation arising from the underlying clonal hematopoiesis. This phenomenon appears to be particularly prominent in selected MDS subtypes and may represent a distinctive clinical feature of these patients [2]. For MDS patients presenting with fever of unknown origin (FUO) as the initial or predominant manifestation, the diagnostic process is often challenging. Patients frequently undergo repeated empirical antibiotic courses, with prolonged exposure to broad-spectrum agents, before MDS is recognized [2,3]. Unlike infectious fever, MDS-related fever typically responds well to anti-inflammatory agents such as corticosteroids [4,5]. In contrast, indiscriminate antimicrobial use not only fails to control the fever and delays diagnosis but also promotes colonization and infection by multidrug-resistant organisms, thereby increasing the risk of life-threatening infections during subsequent disease-directed therapy [6].

Therefore, identifying clinical features and specific biomarkers that enable early differentiation of MDS-associated FUO from other febrile causes has substantial clinical implications for reducing diagnostic delays, curbing antibiotic overuse, and facilitating timely initiation of targeted therapy [7]. This study aimed to describe the clinical and laboratory profiles of patients with MDS and FUO, and to explore factors associated with in-hospital fever resolution, with the goal of informing the clinical characterization of this patient subset.

## 2. Methods

### 2.1. Study Design and Participants

This was a retrospective, single-center observational study designed to systematically evaluate the clinical and laboratory characteristics of patients with MDS and FUO and to explore factors associated with in-hospital fever resolution. Patients hospitalized at Peking Union Medical College Hospital between 1 January 2013, and 31 December 2022, were screened for enrollment. Inclusion criteria were: (1) admission with a diagnosis of FUO or development of FUO during hospitalization; and (2) MDS as the primary discharge diagnosis. MDS was diagnosed by hematologists according to the World Health Organization classification criteria. FUO was defined as fever with oral temperature > 38.3 °C lasting for at least 3 weeks, with at least three documented febrile episodes, that remained undiagnosed after at least one week of systematic investigation, including blood cultures, autoimmune antibody panels, and imaging studies [8]. The study population comprised two sorts of patients with MDS and FUO. The first group included patients who were diagnosed with MDS during the evaluation of FUO, in whom FUO was the initial manifestation of MDS. The other group included patients with a previously established diagnosis of MDS who were subsequently admitted because of FUO.

All enrolled patients underwent comprehensive evaluation for infectious, neoplastic, and autoimmune etiologies during hospitalization, and the final attribution of fever was confirmed by infectious disease specialists. The final attribution of fever was determined by a multidisciplinary team including infectious disease specialists and hematologists, based on the following: (1) absence of microbiological evidence of infection, including negative blood cultures, negative cultures from other sterile sites when clinically indicated, and negative T-SPOT.TB or no evidence of active tuberculosis on imaging; (2) absence of radiological evidence of infection on chest CT and other imaging as clinically indicated; (3) lack of fever resolution despite adequate empirical antimicrobial therapy; and (4) fever resolution after anti-inflammatory or immunosuppressive therapy, or spontaneous resolution in the absence of anti-infective therapy. Coexisting infections, when identified, were documented, and patients with documented active infection were not classified as having MDS-attributable fever unless fever persisted despite appropriate anti-infective treatment. Clinical data were independently extracted by two infectious disease physicians, with disagreements resolved by a third senior expert.

Based on clinical outcomes following treatment during hospitalization, the MDS FUO cohort was divided into a treatment-responsive group and a treatment-unresponsive group. Treatment response was defined as normalization of body temperature (≤37.3 °C) sustained for at least 72 h, together with either a reduction of high-sensitivity C-reactive protein (hsCRP) by ≥50% from the pre-treatment peak value or a return of hsCRP to within the normal reference range (<8 mg/L), in the absence of fever recurrence during hospitalization. Treatment unresponsiveness was defined as recurrent fever, lack of improvement or progressive elevation of inflammatory markers as judged by the treating physicians, or death during hospitalization [9]. As this was a retrospective study, these definitions were based on documented clinical and laboratory assessments rather than on pre-specified numerical thresholds.

### 2.2. Data Collection

The following data were extracted from the electronic medical record system:

(1) Baseline data: age, sex, and past medical history. For patients with an established MDS diagnosis before admission, MDS subtype and previous bone marrow examination results were also collected.

(2) Clinical data: duration of fever, peak temperature, accompanying symptoms, and use of antibiotics, glucocorticoids, antifungal agents, and empirical anti-tuberculosis therapy prior to admission.

(3) Laboratory parameters: The lowest values recorded during the hospitalization course were collected for hemoglobin (HGBmin), platelet count (PLTmin), and white blood cell count (WBCmin). The highest values recorded during the hospitalization course were collected for high-sensitivity CRP (hsCRPmax), erythrocyte sedimentation rate (ESRmax), and serum ferritin (SFmax), to capture the most severe inflammatory status during the illness. Additional parameters, including lactate dehydrogenase (LDH), peripheral blood T lymphocyte subsets (CD8+CD38+ and CD8+HLA DR+, measured by flow cytometry), tumor markers, autoantibodies (including antinuclear antibodies, anti-double-stranded DNA, extractable nuclear antigen antibodies, anti-neutrophil cytoplasmic antibodies, and antiphospholipid antibodies), and T-cell spot test for tuberculosis (T-SPOT.TB), were all obtained before the initiation of glucocorticoids or immunosuppressants. For patients diagnosed with MDS during hospitalization, bone marrow smear and cytogenetic analysis results were collected, and the revised International Prognostic Scoring System (IPSS-R) score was calculated accordingly. IPSS-R scoring was performed only for patients who had completed bone marrow cytogenetic testing; these data were available in 65 of 89 patients, and percentages were calculated using 65 as the denominator.

(4) Treatment information: use of antibiotics, glucocorticoids, and immunosuppressive agents during hospitalization, as well as clinical outcomes after treatment. Glucocorticoid dose categories were defined as follows: low-to-moderate dose, prednisone-equivalent <0.5 mg/kg/day or methylprednisolone <30 mg/day; full dose, prednisone-equivalent 0.5–1 mg/kg/day or methylprednisolone 40–80 mg/day [10]. Immunosuppressive agents included cyclosporine, tacrolimus, mycophenolate mofetil, and thalidomide, administered orally.

### 2.3. Statistical Analysis

Statistical analyses were performed using SPSS version 26.0 (IBM Corp, Armonk, NY, USA). All continuous variables were presented as median with interquartile range (IQR). Categorical variables were presented as frequencies and percentages (n, %). For intergroup comparisons, continuous variables were analyzed using the Mann–Whitney U test. Categorical variables were compared using Fisher’s exact test.

Given the exploratory, descriptive nature of this study, univariable binary logistic regression was performed to explore variables associated with in-hospital fever response (treatment-responsive vs. treatment-unresponsive), with treatment response as the dependent variable. Variables included age, sex, MDS subtype, mucocutaneous lesions, CD8+CD38+ T-cell percentage, glucocorticoid use, immunosuppressant use, and IPSS-R. Results are reported as odds ratios (ORs) with 95% confidence intervals (CIs). Because of the limited number of treatment-responsive events (n = 37) and the resulting instability of the multivariable model, only univariable results are presented. *p*-values should be interpreted as descriptive indicators of association rather than as formal tests of inference.

In this study, some laboratory parameters, including T lymphocyte subsets and bone marrow cytogenetics, were missing in a proportion of cases, as marked in the results. Given the low proportion of missingness and the assumption that data were missing at random, we adopted a complete case analysis approach, analyzing only cases with complete data for each respective variable without imputation. No adjustment for multiple comparisons was applied, as all analyses in this study were considered exploratory in nature.

### 2.4. Use of AI-Assisted Tools

AI-assisted tools were used solely for language editing and proofreading of the manuscript. They were not used for data analysis, interpretation of results, or generation of scientific content.

## 3. Results

### 3.1. Baseline Characteristics and Pre-Admission Status of MDS FUO Patients

Based on the diagnostic and inclusion criteria described above, a total of 98 patients with MDS and FUO were initially identified. Among these, nine were excluded: five due to insufficient bone marrow data to confirm MDS, three due to insufficient duration of fever to fulfill the FUO definition, and one due to severely incomplete medical records. Ultimately, 89 patients with MDS and FUO were included in the final analysis (enrollment rate: 90.8%).

According to the clinical outcomes, the 89 MDS patients with FUO were divided into a treatment-responsive group (n = 37) and a treatment-unresponsive group (n = 52). As shown in Table 1, the two groups were comparable with respect to age, sex, and medical history.

In this selected cohort of patients with both MDS and FUO, 70 of 89 patients (78.7%) were diagnosed with MDS during the evaluation of FUO, whereas 19 patients (21.3%) had a pre-existing MDS diagnosis and subsequently developed FUO. This proportion reflects the selection criteria of the present study and should not be interpreted as the prevalence of FUO among all patients with MDS. Over half of the patients had high or very high risk according to the IPSS-R scores. In terms of cytogenetic abnormalities, complex karyotype abnormalities were identified in 25.8% (23/89) of patients, and trisomy 8 was the most common single abnormality, accounting for 15.7% (14/89). None of these baseline characteristics differed significantly between the two groups, indicating that the groups were comparable in terms of baseline disease severity and cytogenetic risk profiles.

Notably, the distribution of MDS subtypes differed significantly between the two groups (*p* = 0.027). The treatment-responsive group had a higher proportion of Lower-blast MDS (MDS-LB) (70.3% vs. 48.1%), whereas increased-blast MDS (MDS-IB) was more frequent in the treatment-unresponsive group (16.2% vs. 42.3%).

Detailed comparisons of clinical features between the two groups were presented in Table 2. The median duration of fever was 3 months, and the majority of patients presented with high fever exceeding 39 °C, with similar fever patterns (*p* = 0.999) and durations (*p* = 0.799) between groups. Most clinical symptoms did not differ significantly between the two groups, including the digestive system (abdominal pain, diarrhea, intestinal obstruction, perianal lesions, etc.), respiratory system (cough, pleural effusion, pulmonary infiltrates, etc.), and osteoarticular system (polyarthralgia). Mucocutaneous lesions, however, including oral or genital ulcers, various skin rashes, erythema nodosum, and subcutaneous nodules, were observed in a subset of patients, with some cases confirmed as Sweet syndrome by histopathological examination. Notably, the proportion of patients with mucocutaneous lesions was significantly higher in the treatment-responsive group than in the unresponsive group (54.1% vs. 21.2%, *p* = 0.002).

A total of 84.3% (75/89) of patients had received empirical antibiotic therapy before hospitalization after fever onset, with over half (56.2%, 50/89) having been treated by broad-spectrum antimicrobial agents. In addition, 43.8% (39/89) of patients had received glucocorticoid therapy prior to admission, although predominantly at low-to-moderate doses and on an intermittent basis. A total of 21.3% (19/89) of patients had received immunosuppressive agents. The utilization of these therapeutic categories did not differ significantly between the treatment-responsive and treatment-unresponsive groups (all *p*-values exceeded 0.05), suggesting that empirical pre-admission treatments, including suboptimal glucocorticoid regimens, failed to improve clinical outcomes.

### 3.2. Post-Admission Laboratory Findings and Diagnosis

Comparisons of laboratory parameters between the two groups are summarized in Table 3. Most laboratory indices did not differ significantly between the treatment-responsive and treatment-unresponsive groups, with the exception of the CD8+CD38+T-cell percentage and T-SPOT.TB positivity rate, which showed statistically significant differences. Among them, CD8+CD38+ T-cell percentage was available in 37 of 89 patients (41.6%), including 20 in the treatment-responsive group and 17 in the treatment-unresponsive group, and all patients underwent T-SPOT.TB test.

Regarding etiological assessment, systematic evaluation revealed no definitive infectious or pathogenic evidence in the vast majority of patients. Although a small proportion of patients had coexisting infections, antimicrobial therapy alone did not effectively control fever. These findings suggested that the prolonged fever in this cohort was primarily attributable to MDS-related inflammatory processes rather than to infection alone.

### 3.3. Treatment Details and Clinical Outcomes During Hospitalization

Details of treatments during hospitalization were summarized in Table 4. Over half (55/89) of patients received initial antimicrobial therapy during hospital stay. However, no significant differences were observed between the two groups regarding the use of broad-spectrum antibiotics, including carbapenems and agents with activity against drug-resistant Gram-positive cocci.

In contrast, standardized use of glucocorticoids and immunosuppressive agents was associated with fever resolution in this retrospective cohort. The proportion of patients receiving full-dose glucocorticoid therapy was higher in the treatment-responsive group than in the treatment-unresponsive group (*p* = 0.005). Similarly, the proportion of patients receiving standardized immunosuppressive therapy was higher in the treatment-responsive group (*p* = 0.043).

### 3.4. Factors Associated with In-Hospital Fever Resolution

As shown in Table 5, univariable binary logistic regression analysis identified mucocutaneous lesions (OR 4.39, 95% CI 1.73–11.09; *p* = 0.002), glucocorticoid use (OR 4.40, 95% CI 1.77–10.91; *p* = 0.001), immunosuppressant use (OR 2.98, 95% CI 1.08–8.19; *p* = 0.034), and higher CD8+CD38+ T-cell percentage (per 1% increase: OR 1.06, 95% CI 1.01–1.11; *p* = 0.011) were associated with in-hospital fever resolution. MDS-IB subtype was associated with a lower likelihood of fever response compared with MDS-LB or other subtypes (OR 0.26, 95% CI 0.09–0.74; *p* = 0.011). Age (OR 0.99, 95% CI 0.97–1.02; *p* = 0.494) and sex (male vs. female: OR 1.25, 95% CI 0.52–3.00; *p* = 0.616) were not associated with fever response. Because of the limited number of treatment-responsive events (n = 37), a multivariable model could not be stably estimated, and only univariable results are presented. These findings should be regarded as exploratory and hypothesis-generating.

## 4. Discussion

Patients with MDS and FUO pose substantial diagnostic challenges in clinical practice. Distinguishing MDS-related FUO from infectious FUO is often difficult, as both can manifest as prolonged fever, elevated inflammatory markers, and constitutional symptoms [11]. This diagnostic ambiguity frequently leads to prolonged and multiple empirical antimicrobial courses, increasing risks of unnecessary drug exposure and delayed diagnosis and treatment. In the present study, we systematically synthesize the clinical, laboratory, and therapeutic characteristics of MDS patients presenting with FUO to improve clinicians’ recognition of this challenging condition. Our study indicated that MDS patients with FUO were predominantly at high risk and carried unfavorable disease-related factors. Multilineage cytopenias and markedly elevated ferritin levels were the most significant laboratory findings; moreover, these patients showed poor response to antimicrobials but had a favorable response to corticosteroids.

MDS includes a highly heterogeneous disease spectrum with various presentations and prognoses. Our findings showed that the IPSS-R scores of patients with MDS-driven FUO were generally high, with more than half falling into the high-risk category and a substantial proportion having complex karyotypes, consistent with previous reports [12]. Although these factors were not statistically associated with in-hospital fever resolution, they reflect the overall high-risk disease profile of this patient subset, which should be noted when caring for such patients. Previous studies suggested that autoimmune complications may not be strongly correlated with MDS subtypes [4], while others reported higher proportions of single lineage dysplasia MDS (MDS-SLD) among MDS patients with immune-related manifestations [13]. In our cohort, the treatment-responsive group had a higher proportion of MDS-LB, whereas the treatment-unresponsive group had a higher proportion of MDS-IB. This difference may be explained by the fact that MDS-IB is characterized by a significant increase in blast cells, which generally indicates more severe impairment of bone marrow hematopoiesis and a higher risk of disease progression [14]. These patients may be more prone to persistent inflammatory status, show relatively poor clinical response to immunosuppressive therapy, and face higher risks of infectious complications following treatment. In contrast, MDS-LB follows a relatively indolent clinical course, with better treatment tolerance and lower risks of infectious complications during immunosuppressive therapy, which may contribute to more favorable in-hospital fever response.

The most prominent clue indicating MDS-associated FUO was marked cytopenia. In the present cohort, over 90% of patients had bicytopenia or pancytopenia, and over half had pancytopenia. Of note, 78.7% of the patients in this cohort received their MDS diagnosis only after the onset of fever, and cytopenia often occurred concurrently with or even preceded the fever, suggesting that cytopenia may serve as the initial laboratory abnormality. This highlights that when evaluating the cause of FUO, abnormal blood counts, particularly multilineage cytopenia, should raise suspicion for underlying hematologic disorders such as MDS, rather than being automatically attributed to inflammatory consumption or severe infection. In such circumstances, bone marrow examination should be considered promptly [3].

In addition, inflammatory markers were markedly elevated in MDS patients with FUO, with serum ferritin showing the most prominent increase. Although most inflammatory parameters did not differ significantly between the two outcome groups, they tended to be higher in the unresponsive group, suggesting that persistent inflammation may be associated with protracted fever and poorer outcomes. As is known, ferritin is not merely an indicator of body iron stores but also an important acute-phase protein. Its elevation often signals inflammatory activation, macrophage system dysregulation, and cytokine-mediated immune imbalance. Previous studies have shown that a chronic inflammatory microenvironment exists in MDS, with aberrant expression of pro-inflammatory cytokines such as IL 6 and TNF α, which may contribute to ineffective hematopoiesis, bone marrow failure, and infection susceptibility [15]. Thus, marked ferritin elevation in these patients may also reflect activation of MDS-related inflammatory pathways. Of note, the consistently higher inflammatory markers observed in the treatment-unresponsive group suggest that persistent inflammation is linked to prolonged disease course or poor treatment response in MDS patients with FUO. Nevertheless, the diagnostic and prognostic value of these markers warrants further validation in larger prospective cohorts; transfusion history and iron overload status were not systematically collected, and ferritin elevation may partly reflect iron overload rather than inflammation alone.

Interestingly, the treatment-unresponsive group exhibited a higher T SPOT.TB positivity rate in our study. This assay measures the release of interferon gamma from T-cells in response to specific *Mycobacterium tuberculosis* antigen stimulation, and is therefore an antigen-specific assay; its positivity reflects the magnitude of antigen-specific T-cell immune activation and indicates prior or current exposure to *M. tuberculosis*, but does not by itself diagnose active tuberculosis [16]. In this cohort, patients with a positive T-SPOT.TB result were evaluated for active tuberculosis by clinical presentation, imaging, and microbiological tests, and active tuberculosis was diagnosed in only 2 patients. The higher T-SPOT.TB positivity rate in the treatment-unresponsive group may therefore reflect a higher prevalence of latent tuberculosis infection or prior exposure in this subgroup, or a greater degree of antigen-specific T-cell immune activation associated with more severe inflammation, rather than active tuberculosis. Importantly, a positive T-SPOT.TB result should not be interpreted as evidence of active tuberculosis in patients with MDS and FUO; it should be evaluated together with clinical presentation, imaging findings, and other microbiological evidence to avoid misdiagnosis and unnecessary anti-tuberculosis therapy.

Although data on neutropenia were not systematically collected in the present analysis, coexisting infections were documented in some patients, and fever did not resolve substantially following appropriate antimicrobial therapy. These findings suggest that infection alone was unlikely to account for the persistent fever in this cohort, and that MDS-related inflammation may have contributed to the febrile state. Nevertheless, we cannot completely exclude occult or culture-negative infection, particularly in patients with severe neutropenia. Persistent fever in neutropenic patients requires continued evaluation for infection, and the absence of ANC and neutropenia duration data is an important limitation of this study.

Lymphocyte immunophenotyping analysis suggested that both CD8+CD38+ and CD8+HLA DR+ T-cell percentages were higher in the treatment-responsive group than in the treatment-unresponsive group, but only the difference in CD8+CD38+ T-cell percentage reached statistical significance. Both CD8+CD38+ and CD8+HLA DR+ T-cells are activated T lymphocyte subsets and typically increase upon activation of cellular immunity [7]. However, patients with MDS commonly exhibit reduced numbers and functional impairment of CD8+ T-cells, reflecting an immune exhaustion state [17]. In this context, the elevated CD8+CD38+ T-cell percentage observed in the treatment-responsive group suggests relatively less T lymphocyte exhaustion and better-preserved immune surveillance and response capacity, enabling more effective control of inflammatory imbalance and potential infections, and ultimately better clinical outcomes. This inference aligns with previous findings that MDS patients who respond well to immunosuppressive therapy have higher proportions of activated T-cells [18]. In contrast, CD8+HLA DR+ T-cells may be more susceptible to exhaustion in the MDS bone marrow microenvironment, potentially influenced by myeloid-derived suppressor cells [19], thereby diminishing their predictive value. These observations suggest that CD8+CD38+ T-cell proportion may better reflect functional T lymphocyte reserve and serve as a potential immunological marker for in-hospital fever resolution in MDS patients with FUO.

The treatment findings of this study offer important implications for clinical decision-making. Although MDS patients are at increased risk of infection, fever and inflammatory status could also be rooted in MDS itself. When active infection has been fully ruled out in febrile patients, prompt and standardized use of glucocorticoids and immunosuppressants was significantly associated with fever resolution and favorable clinical outcomes. Further analysis revealed that patients receiving full-dose glucocorticoid therapy had better fever outcomes. Previous studies have suggested that glucocorticoids can serve as first-line therapy for MDS patients with autoimmune manifestations, with favorable responses observed in approximately 80% of patients; however, a considerable proportion of patients may develop steroid dependence, and some may even experience exacerbation of symptoms following glucocorticoid treatment [5]. In terms of immunosuppressants, most clinical practice considers them as second-line agents, but there were also studies showing no survival benefits [20]. Therefore, no consensus has been reached on the optimal management of MDS-related inflammation. Nevertheless, our findings have practical implications. For patients with high suspicion of MDS-related fever, when extensive screening work-up has excluded identifiable infections, clinicians should consider that the etiology of fever is due to underlying disease and initiate anti-inflammatory therapy with glucocorticoids or immunosuppressants. This therapeutic shift may help promptly control systemic inflammation while reducing unnecessary broad-spectrum and prolonged antibiotic exposure, which may in turn reduce the risk of subsequent multidrug-resistant infections. Given the non-randomized design, these associations may be influenced by confounding by indication and should be interpreted as hypothesis-generating. They do not establish causality but are consistent with a possible immune-mediated inflammatory contribution to fever in MDS, which merits further prospective investigation.

In exploratory univariable analyses, mucocutaneous lesions, glucocorticoid use, immunosuppressant use, and higher CD8+CD38+ T-cell percentage were associated with in-hospital fever resolution, whereas MDS-IB subtype was associated with a lower likelihood of response. These findings are consistent with the descriptive comparisons in Table 2 and Table 4 and support the hypothesis that MDS-related fever may have an immune-mediated inflammatory component. However, because of the limited number of treatment-responsive events and the absence of a non-MDS FUO comparison group and long-term follow-up data, these associations should be interpreted as exploratory and cannot be used to establish diagnostic or prognostic markers. Prospective studies with larger sample sizes are needed to validate these findings.

Several limitations should be acknowledged. First, this was a single-center retrospective study with a relatively small sample size, and selection bias and missing data cannot be excluded. Second, the absence of a non-MDS FUO comparison group and of long-term follow-up data precludes any conclusion regarding the diagnostic or prognostic value of the laboratory parameters identified in this study. Third, absolute neutrophil count and duration of neutropenia were not systematically collected, and persistent fever in neutropenic patients requires continued evaluation for infection. Fourth, the retrospective design and lack of a standardized treatment protocol mean that the associations between glucocorticoid or immunosuppressant use and fever response may be confounded by indication. In addition, the laboratory values used were the lowest blood counts and highest inflammatory markers recorded during hospitalization rather than strictly baseline values, and should be interpreted as indicators of the most severe hematological and inflammatory status during the hospital course. Finally, because of the limited number of treatment-responsive events (n = 37), a multivariable logistic regression model could not be stably estimated, and only univariable results are presented; these findings should therefore be regarded as exploratory.

## 5. Conclusions

FUO may represent a clinically important presentation in a subset of patients with MDS. The present study systematically characterizes the clinical and laboratory profiles of patients with MDS presenting as FUO, and found that MDS patients with FUO are predominantly at high risk and carry unfavorable disease-related factors. Laboratory features were dominated by multilineage cytopenias and markedly elevated ferritin levels. T-lymphocyte subset analysis may offer additional value in the clinical evaluation of this patient subset, although its role in differential diagnosis requires further study with non-MDS FUO comparison groups. The poor response to antimicrobials and favorable response to corticosteroids and immunosuppressants are consistent with an immune-mediated inflammatory mechanism for fever in this population. Given the single-center retrospective design of this study, future multicenter prospective studies with larger sample sizes are required to validate our findings and to further elucidate the underlying molecular mechanisms.

## Figures and Tables

**Table 1:** Baseline clinical characteristics of patients with MDS and FUO.

Variables	Overall (N = 89)	Treatment-Responsive Group (n = 37)	Treatment-Unresponsive Group (n = 52)	*p*-Value
Sex [n (%)]				0.663
Male	55 (61.8%)	24 (64.9%)	31 (59.6%)	
Female	34 (38.2%)	13 (35.1%)	21 (40.4%)	
Age [years, median (IQR)]	54 (41, 65)	53 (41, 62)	57 (43.3, 65.3)	0.464
Comorbidities [n (%)]				
Diabetes mellitus	22 (24.7%)	8 (21.6%)	14 (26.9%)	0.625
Malignancy	5 (5.6%)	0 (0.0%)	5 (9.6%)	0.073
Autoimmune diseases	11 (12.4%)	4 (10.8%)	7 (13.5%)	0.757
Chronic infection	6 (6.7%)	3 (8.1%)	3 (5.8%)	0.690
MDS diagnosed before fever onset	19 (21.3%)	10 (27.0%)	9 (17.3%)	0.303
Time from pre-existing MDS diagnosis to fever [months, median (IQR)]	24 (8, 108)	24 (9, 36)	12 (7, 108)	0.712
MDS subtype [n (%)]				**0.027**
MDS-IB	28 (31.5%)	6 (16.2%)	22 (42.3%)	
MDS-LB	51 (57.3%)	26 (70.3%)	25 (48.1%)	
Others	10 (11.2%)	5 (13.5%)	5 (9.6%)	
IPSS-R Score [n (%)]				0.855
Very low	1 (1.5%)	1 (3.7%)	0	
Low	4 (6.2%)	1 (3.7%)	3 (7.9%)	
Intermediate	14 (21.5%)	6 (22.2%)	8 (21.1%)	
High	19 (29.2%)	8 (29.6%)	11 (28.9%)	
Very high	27 (41.5%)	11 (40.7%)	16 (42.1%)	
Cytogenetic abnormalities [n (%)]				0.845
Trisomy 8	14 (15.7%)	6 (16.2%)	8 (15.4%)	
Complex karyotype (≥3 abnormalities)	23 (25.8%)	11 (29.7%)	12 (23.1%)	
Other abnormalities	19 (21.3%)	7 (18.9%)	12 (23.1%)	
Normal karyotype	9 (10.1%)	3 (8.1%)	6 (11.5%)	

MDS, myelodysplastic syndrome; MDS-IB, increased-blast myelodysplastic syndrome; MDS-LB, lower-blast myelodysplastic syndrome; IPSS-R, revised International Prognostic Scoring System; IQR, interquartile range. Bold *p* values indicate statistical significance (*p* < 0.05).

**Table 2:** Clinical characteristics and pre-admission medications of the study patients with different causes.

Variables	Overall (N = 89)	Treatment-Responsive Group (n = 37)	Treatment-Unresponsive Group (n = 52)	*p*-Value
Clinical manifestations				
Fever duration [months, median (IQR)]	3 (1, 8)	3 (1, 6)	3 (1, 10.75)	0.799
High fever (>39 °C) [n (%)]	77 (86.5%)	32 (86.5%)	45 (86.5%)	0.999
Accompanying symptoms [n (%)]				
Gastrointestinal involvement	26 (29.2%)	12 (32.4%)	14 (26.9%)	0.640
Respiratory involvement	20 (22.5%)	6 (16.2%)	14 (26.9%)	0.306
Mucocutaneous lesions	31 (34.8%)	20 (54.1%)	11 (21.2%)	**0.002**
Osteoarticular involvement	21 (23.6%)	11 (29.7%)	10 (19.2%)	0.313
Pre-admission medications				
Antibiotics				0.956
None	14 (15.7%)	6 (16.2%)	8 (15.4%)	
Conventional antibiotics	25 (28.1%)	11 (29.7%)	14 (26.9%)	
Broad-spectrum antibiotics	50 (56.2%)	20 (54.1%)	30 (57.7%)	
Antifungal treatment [n (%)]	15 (16.9%)	6 (16.2%)	9 (17.3%)	0.998
Empirical anti-tuberculosis therapy [n (%)]	16 (18.0%)	7 (18.9%)	9 (17.3%)	0.989
Glucocorticoid use				0.675
None	50 (56.2%)	19 (51.4%)	31 (59.6%)	
Low-to-moderate dose	20 (22.5%)	10 (27.0%)	10 (19.2%)	
Full dose	19 (21.3%)	8 (21.6%)	11 (21.2%)	
Immunosuppressants	19 (21.3%)	9 (24.3%)	10 (19.2%)	0.607

MDS, myelodysplastic syndrome; FUO, fever of unknown origin. Bold *p* values indicate statistical significance (*p* < 0.05).

**Table 3:** Post-admission laboratory findings and diagnosis of patients with MDS and FUO.

Variables	Overall (N = 89)	Treatment-Responsive Group (n = 37)	Treatment-Unresponsive Group (n = 52)	*p*-Value
Complete blood count				
HGBmin [g/L, median (IQR)]	62 (54, 70)	63 (58, 70)	62 (54, 69)	0.487
PLTmin [×10^9^/L, median (IQR)]	43 (17, 100)	45 (22, 87)	40 (17, 101)	0.745
WBCmin [×10^9^/L, median (IQR)]	2.00 (1.44, 3.10)	2.23 (1.48, 3.25)	1.91 (1.39, 2.85)	0.292
Number of cytopenic lineages				0.655
Single lineage cytopenia	6 (6.7%)	3 (8.1%)	3 (5.8%)	
Bicytopenia	34 (38.3%)	12 (32.4%)	22 (42.3%)	
Pancytopenia	49 (55.1%)	22 (59.5%)	27 (51.9%)	
Inflammatory markers				
hsCRPmax [mg/L, median (IQR)]	123.6 (63.8, 162.0)	107.0 (73.5, 140.0)	140.7 (64.4, 184.9)	0.258
ESRmax [mm/H, median (IQR)]	114 (81, 140)	111 (74, 140)	114 (86, 140)	0.603
SFmax [ng/ml, median (IQR)]	1061 (620, 1500)	1132 (651, 1765)	1134 (536, 1500)	0.438
LDH [U/L, median (IQR)]	277 (185, 449)	293 (191, 398)	277 (184, 534)	0.653
T-lymphocyte subset analysis				
CD4+T-cell count [cells/μL, median (IQR)] (n = 46)	357 (168, 656)	359 (172, 656) (n = 26)	357 (212, 502) (n = 20)	0.965
CD8+DR+T-cell percentage [%, median (IQR)] (n = 36)	41.7 (28.8, 56.3)	44.0 (32.8, 64.0) (n = 20)	37.0 (22.6, 52.8) (n = 16)	0.152
CD8+CD38+T-cell percentage [%, median (IQR)] (n = 37)	58.8 (44.1, 75.3)	66.7 (60.4, 80.6) (n = 20)	47.4 (38.5, 57.7) (n = 17)	**0.003**
Other tests				
Tumor markers [n (%)]	17 (19.1%)	7 (18.9%)	10 (19.2%)	0.716
Autoantibodies [n (%)]	19 (21.3%)	5 (13.5%)	14 (26.9%)	0.067
T-SPOT.TB [n (%)]	22 (24.7%)	6 (16.2%)	16 (30.8%)	**0.034**
Coexisting infections				0.751
None	67 (75.3%)	29 (78.4%)	38 (73.1%)	
Bacterial infection	15 (16.9%)	6 (16.2%)	9 (17.3%)	
Tuberculosis	2 (2.2%)	1 (2.7%)	1 (1.9%)	
Fungal infection	5 (5.6%)	1 (2.7%)	4 (7.7%)	

HGBmin, lowest hemoglobin; PLTmin, lowest platelet count; WBCmin, lowest white blood cell count; hsCRPmax, highest high-sensitivity C-reactive protein; ESRmax, highest erythrocyte sedimentation rate; SFmax, highest serum ferritin; LDH, lactate dehydrogenase; T-SPOT.TB, T-cell spot test for tuberculosis; IQR, interquartile range. Bold *p* values indicate statistical significance (*p* < 0.05).

**Table 4:** Treatment details and clinical outcomes during hospitalization of patients with MDS and FUO.

Variables	Overall (N = 89)	Treatment-Responsive Group (n = 37)	Treatment-Unresponsive Group (n = 52)	*p*-Value
Antibiotics [n (%)]				0.786
None	34 (38.2%)	15 (40.5%)	19 (36.5%)	
Conventional antibiotics	18 (20.2%)	6 (16.2%)	12 (23.1%)	
Broad-spectrum antibiotics	37 (41.6%)	16 (43.2%)	21 (40.4%)	
Glucocorticoid use [n (%)]				**0.004**
None	26 (29.2%)	6 (16.2%)	20 (38.5%)	
Low to moderate dose	28 (31.5%)	9 (24.3%)	19 (36.5%)	
Full dose	35 (39.3%)	22 (59.5%)	13 (25.0%)	
Immunosuppressants [n (%)]	21 (23.6%)	13 (35.1%)	8 (15.4%)	**0.043**

MDS, myelodysplastic syndrome; FUO, fever of unknown origin. Bold *p* values indicate statistical significance (*p* < 0.05).

**Table 5:** Univariable binary logistic regression analysis of factors associated with in-hospital fever response in patients with MDS and FUO.

Variables	OR (95%CI)	*p*-Value
Age (per year)	0.99 (0.97–1.02)	0.494
Sex (male vs. female)	1.25 (0.52–3.00)	0.616
MDS-IB vs. MDS-LB/others	0.26 (0.09–0.74)	**0.011**
Mucocutaneous lesions (yes vs. no)	4.39 (1.73–11.09)	**0.002**
CD8+CD38+ T-cells (per 1%)	1.06 (1.01–1.11)	**0.011**
Glucocorticoid use (Full dose vs. others)	4.40 (1.77–10.91)	**0.001**
Immunosuppressant use (yes vs. no)	2.98 (1.08–8.19)	**0.034**

OR, odds ratio; CI, confidence interval; MDS-IB, increased-blast myelodysplastic syndrome; MDS-LB, lower-blast myelodysplastic syndrome. Treatment response (treatment-responsive vs. treatment-unresponsive) was the dependent variable. Because of the limited number of treatment-responsive events (n = 37), a multivariable model could not be stably estimated; therefore, only univariable results are presented. *p*-values are descriptive and were not adjusted for multiple comparisons. Bold *p* values indicate statistical significance (*p* < 0.05).

## Data Availability

The datasets analyzed during the current study are not publicly available because the database is still under ongoing analysis, but are available from the corresponding author upon reasonable request.

## Abbreviations

FUO, fever of unknown origin; MDS, myelodysplastic syndrome; IPSS-R, revised International Prognostic Scoring System; MDS-LB, lower-blast myelodysplastic syndrome; MDS-IB, increased-blast myelodysplastic syndrome; CRP, C-reactive protein; LDH, lactate dehydrogenase; T-SPOT.TB, T-cell spot test for tuberculosis; IQR, interquartile range; MDS-SLD, single lineage dysplasia myelodysplastic syndrome; ORs, odds ratios; CIs, confidence intervals.
